# Technology-aided programs for post-coma patients emerged from or in a minimally conscious state

**DOI:** 10.3389/fnhum.2014.00931

**Published:** 2014-12-05

**Authors:** Giulio E. Lancioni, Nirbhay N. Singh, Mark F. O’Reilly, Jeff Sigafoos, Marta Olivetti Belardinelli, Francesca Buonocunto, Fiora D’Amico, Jorge Navarro, Crocifissa Lanzilotti, Gabriele Ferlisi, Floriana Denitto

**Affiliations:** ^1^Department of Neuroscience and Sense Organs, University of BariBari, Italy; ^2^Medical College of Georgia, Georgia Regents UniversityAugusta, GA, USA; ^3^Department of Special Education, University of Texas at AustinAustin, TX, USA; ^4^Department of Educational Psychology, Victoria University of WellingtonWellington, New Zealand; ^5^Department of Psychology, “Sapienza” University of RomeRome, Italy; ^6^S. Raffaele Rehabilitation and Care CentersCeglie and Alberobello, Italy; ^7^Lega F. D’Oro Research CenterOsimo, Italy; ^8^ISPE Medical Care CenterMola di Bari, Bari, Italy

**Keywords:** technology-aided programs, minimally conscious state (MCS), emergence from MCS, leisure stimuli, social stimuli, news, choice

## Abstract

Post-coma persons in a minimally conscious state (MCS) or emerged/emerging from such state (E-MCS), who are affected by extensive motor impairment and lack of speech, may develop an active role and interact with their environment with the help of technology-aided intervention programs. Although a number of studies have been conducted in this area during the last few years, new evidence about the efficacy of those programs is warranted. These three studies were an effort in that direction. Study I assessed a technology-aided program to enable six MCS participants to access preferred environmental stimulation independently. Studies II and III assessed technology-aided programs to enable six E-MCS participants to make choices. In Study II, three of those participants were led to choose among leisure and social stimuli, and caregiver interventions automatically presented to them. In Study III, the remaining three participants were led to choose (a) among general stimulus/intervention options (e.g., songs, video-recordings of family members, and caregiver interventions); and then (b) among variants of those options. The results of all three studies were largely positive with substantial increases of independent stimulation access for the participants of Study I and independent choice behavior for the participants of Studies II and III. The results were analyzed in relation to previous data and in terms of their implications for daily contexts working with MCS and E-MCS persons affected by multiple disabilities.

## Introduction

Post-coma persons in a minimally conscious state (MCS) or emerged/emerging from such a state (E-MCS), and affected by extensive motor impairment and lack of speech, are typically unable to manage interactions with their context and regulate their stimulation input (i.e., determine the amount and types of stimulation available to them) (Giacino, 1996; Elliott and Walker, 2005; Katz et al., 2009; Nakase-Richardson et al., 2009; Lancioni et al., 2010; Bruno et al., 2011; Giacino et al., 2012; de Jong, 2013; Eifert et al., 2013). Ensuring that they receive sufficient stimulation is considered a priority of rehabilitation and care centers or family contexts responsible for their wellbeing (Whyte, 2007; Hirschberg and Giacino, 2011; Conneeley, 2012; McNamee et al., 2012; Noé et al., 2012; Müller-Patz et al., 2013; Seel et al., 2013).

Stimulation (environmental enrichment) programs available for such purpose (Giacino, 1996; Vanier et al., 2002; Barreca et al., 2003; Bekinschtein et al., 2005; Elliott and Walker, 2005; Whyte, 2007; Zhu et al., 2009; Lotze et al., 2011) might be only partially satisfactory. In fact, such programs do not allow the persons to have an active role and do not ensure that they receive the amount or type of stimulation they find preferable (Lancioni et al., 2014b). Technology-aided intervention programs (i.e., procedures based on the use of assistive technology to monitor participants’ responding and make it instrumental to access or choose among stimulation and social or communication events) may be a valuable and affordable alternative to the stimulation/enrichment approaches (Lancioni et al., 2010, 2011a,b; Wallace and Bradshaw, 2011; Baxter et al., 2012; de Joode et al., 2012; Scherer, 2012). Indeed, technology-aided programs (a) enable the persons to acquire an active role and exercise self-determination and choice (thus representing a change of direction compared to the stimulation/enrichment approaches that make the person a basically passive recipient of environmental input); and (b) can be developed/arranged with relatively modest economical costs and with moderate staff time investments (Magee, 2005, 2007; Daveson, 2010; Georgiopoulos et al., 2010; Hirschberg and Giacino, 2011; Lotze et al., 2011; Di Stefano et al., 2012; Naci et al., 2012; Särkämö and Soto, 2012; Lancioni et al., 2014b).

Research with MCS and E-MCS persons, who are affected by extensive motor impairment and lack of speech, has emphasized the usability and beneficial effects of technology-aided programs for supporting, among others, (a) independent access to programed environmental stimuli; and (b) choice among various types of stimuli and caregiver interventions (i.e., with the possibility of selecting and accessing the most preferred options) (Lancioni et al., 2014b). For example, Lancioni et al. (2012b) showed that five MCS participants of 37–78 years of age learned to access stimulation independently with protracted eyelid closures, small hand closures, or toe movement. The participants’ responses were monitored via an optic microswitch on the cheekbone, a touch-pressure microswitch in the hand, and a tilt microswitch on the toe, respectively. The same authors (i.e., Lancioni et al., 2012b) also showed that three E-MCS participants of 49–84 years of age learned to choose among leisure stimuli as well as social stimuli and caregiver interventions. A computer system presented verbally or verbally and visually brief samples of each of the stimuli/interventions available and the participants could choose any of them (i.e., by activating a pressure microswitch immediately after the related sample presentation) or bypass it (i.e., by abstaining from microswitch activation).

Although the results of the aforementioned studies and other studies in the area emphasize the importance of technology-aided programs to promote positive engagement and choice behavior (i.e., skills deemed relevant for the rehabilitation/recovery process), the number of participants involved in the research is relatively small (Lancioni et al., 2011a,b, 2012a, 2013a,e, 2014b). In light of this, new research seems warranted to determine the generality of the results obtained with the technology-aided programs available (Kennedy, 2005; Barlow et al., 2009; Posatskiy and Chau, 2012; McNaughton and Light, 2013; Lancioni et al., 2014b). The present studies were an effort in that direction. Study I assessed a technology-aided program to enable six MCS participants to access preferred environmental stimulation independently. Studies II and III assessed technology-aided programs to enable six E-MCS participants to make choices. In Study II, three of those participants were led to choose among leisure and social stimuli, and caregiver interventions automatically presented to them. In Study III, the remaining three participants were led to choose (a) among general stimulus/intervention options (e.g., songs, video-recordings of family members, and caregiver interventions); and then (b) among variants of those options (Lancioni et al., 2011b).

## Study I

### Method

#### Participants

The six participants included three women (Eunice, Gwen, and Coleen) and three men (Harold, Dustin, and Manuel) who were in rehabilitation or care centers and were diagnosed with MCS and pervasive motor impairment following brain injury and coma. While they could not be interviewed about their involvement in the study, this was considered a pleasant experience for them given that (a) the stimuli used during the intervention phases were deemed preferred (enjoyable) (i.e., based on families’ reports; see below); and (b) the presence of these stimuli increased only if their responding rose (see below). Their families had signed a consent form for the study, which had been approved by a scientific and ethics committee.

The participants’ ages, their coma durations, and the intervals between their brain injury and the start of this study are reported in Table 1. They ranged from 38 to 85 years, from 1 to 3 weeks, and from 2 to 118 months, respectively. The participants’ subscale and total scores on the Coma Recovery Scale-Revised (CRS-R; Kalmar and Giacino, 2005) are reported in Table 2. The total scores varied from 9 to 12. The subscale scores indicating MCS are in bold type. Eunice and Gwen had suffered severe falls causing: (a) occipital subdural hematoma and diffuse axonal injury on bilateral cerebellar and left temporo-parietal regions; and (b) extensive left fronto-temporo-parietal and right temporo-parietal subdural hematomas, respectively. Coleen, Harold, and Dustin had been involved in road accidents with consequent (a) left fronto-temporo-parietal subdural hematoma and diffuse axonal injury on the right and left frontal regions and corpus callosum; (b) left fronto-temporal subdural hematoma and diffuse axonal injury on the fronto-temporal and mesencephalic regions and corpus callosum; and (c) temporo-parieto-occipital subdural hematoma, respectively. Manuel had suffered an extensive left temporo-occipital ischemic stroke.

**Table 1:** **Participants’ ages and coma durations, and intervals between brain injury and start of the study**.

	Participants					
	Eunice	Gwen	Coleen	Harold	Dustin	Manuel
Ages (years)	38	85	51	40	44	75
Coma durations (weeks)	2	2	3	3	2	1
Intervals between brain injury and study (months)	9	10	12	118	2	3

**Table 2:** **Participants’ scores on the CRS-R at the start of the study**.

Subscales	Participants						
	Eunice	Gwen	Coleen	Harold	Dustin	Manuel
Arousal	2	2	2	2	2	2
Oral/Motor	2	2	1	2	2	1
Motor	1	**5**	**3**	2	1	**5**
Communication	0	0	0	**1**	0	0
Visual	**2**	1	1	**2**	**3**	1
Auditory	2	2	2	2	2	2
Total score	9	12	9	11	10	11

*Note: Bold scores indicate minimally conscious state*.

#### Position, responses, technology, and stimuli

The participants were in bed or in a wheelchair during the sessions. The responses selected were present in their behavioral repertoire and appeared comfortable for them (Lancioni et al., 2013b). They consisted of (a) a small lateral movement of the head for Eunice; (b) small finger movements on a flat surface for Gwen; (c) full eyelid closure (blink) for Coleen; (d) prolonged eyelid closure (i.e., longer than 0.8 s) for Harold and Dustin; and (e) repeated eyelid closure (i.e., two blinks within a 2-s interval) for Manuel. The technology involved microswitch devices linked to a computer system. The microswitch devices monitored the participants’ responses and triggered the computer system in connection with their occurrence. The computer system tallied the responses and followed them with brief periods of stimulation (i.e., during the intervention phases of the study; see below). The microswitch for Eunice consisted of an optic sensor involving an infrared light-emitting diode and a mini infrared light-detection unit (Lancioni et al., 2013c). It was attached to a wire fixed at the right side of her neck and pointed to the right side of her chin. Any small rightward movement of the head caused the chin to near the optic sensor thus activating it. The same microswitch was also used for Coleen, Dustin and Manuel. For these participants, the microswitch was fixed with medical tape on the left or right cheekbone (relatively distant from the eye to avoid any visual interference). A mini paper sticker attached to the corresponding eyelid ensured that its closures were reliably recorded by the microswitch (Lancioni et al., 2012c). The microswitch used for Gwen was a 24-cm × 15-cm special touch pad on which Gwen placed her right hand. The pad was divided into quadrants of 2.5 cm × 2.5 cm, which worked as independent touch-sensitive areas. Any finger movement affecting one of the aforementioned areas caused microswitch activation (Lancioni et al., 2012a). The microswitch used for Harold (who was known to dislike anything touching his face) was the Microsoft Kinect sensor (González-Ortega et al., 2014). Its use (supported by the *Just Click* software; B10NIX, Milan, Italy) ensured the recording of Harold’s prolonged eyelid closures from a distance of about 1 m.

During the intervention phases of the study, microswitch activations allowed the participants to access video clips of singing, comedy, old films, prayers, religious hymns, or family events for periods of 10 s. These stimuli were chosen because they included a visual and an auditory component and had been recommended by the participants’ families as representative of participants’ preferences prior to their medical problems. For Coleen, musical videos were initially combined with softly spoken affection words and light stroking of her hands (i.e., in an attempt to increase their impact on responding; see Lancioni et al., 2012a, 2014a).

#### Experimental conditions

Each participant was exposed to an ABAB design, in which A and B represented baseline and intervention phases, respectively (Barlow et al., 2009). The sessions lasted 5 min and were carried out 3–10 times a day depending on the participants’ availability and on their wakefulness (i.e., sessions required that the participants were awake). Data recording concerned the frequencies of responses emitted during the sessions and it was automatically carried out by the computer system. A new response was recorded if at least 10 s had elapsed from the previous response, that is, the 10-s stimulation period for the previous response (or an equivalent period during the A phases) had ended (Lancioni et al., 2012a,c). Response prompting (i.e., a light air puff on the side of the face and a little touch on the face or on the hand) occurred prior to the start of the sessions and during the sessions in case of non-responding for 30–60 s. Research assistants subtracted the responses occurring after prompting from the sessions’ computer tally. Agreement between research assistants on recording these responses (which could also be zero) was reported for each of the 27 sessions in which it was checked.

##### Baseline (A) phases

During these phases, the technology was available to record the responses performed but no stimulation occurred. The phases included 4–15 sessions (i.e., depending on response levels and stability; see Barlow et al., 2009) and ended only if the response frequency of the last session did not exceed that of the previous sessions.

##### Intervention (B) phases

Conditions matched those available during the baseline except that each response was followed by a 10-s period of preferred stimulation. The two phases included 44–63 and 109–211 sessions, respectively. The different lengths of the phases across participants were largely due to the availability of the participants rather than to the time required for establishing or consolidating their responses. In fact, all participants showed clear response increases within relatively short intervention periods and maintained them (see Results). Prior to the start of the first intervention phase, the participants received 4–6 practice sessions, in which the research assistant used prompting to help them experience the relation between responses and stimulation.

### Results

**Figure 1:**
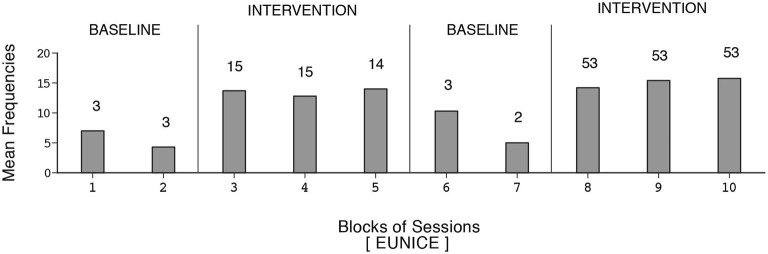
**Eunice’s data**. The bars indicate mean frequencies of responses per session over blocks of sessions. The number of sessions per block/bar is indicated by the numeral above it.

**Figure 2:**
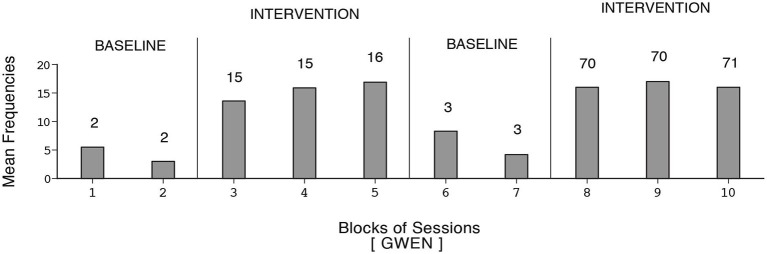
**Gwen’s data plotted as in Figure 1**.

**Figure 3:**
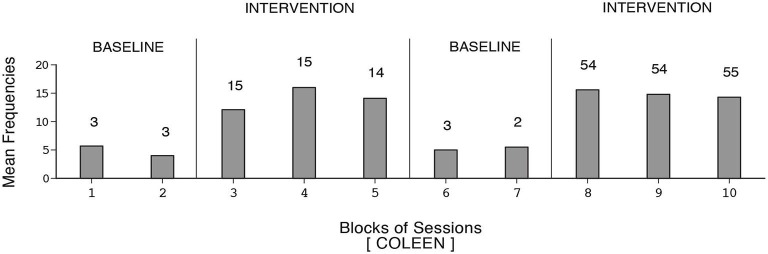
**Coleen’s data plotted as in Figure 1**.

**Figure 4:**
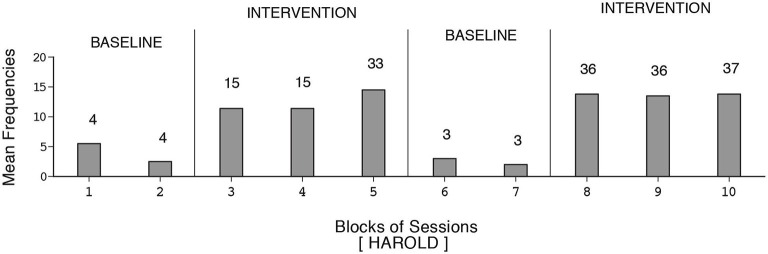
**Harold’s data plotted as in Figure 1**.

**Figure 5:**
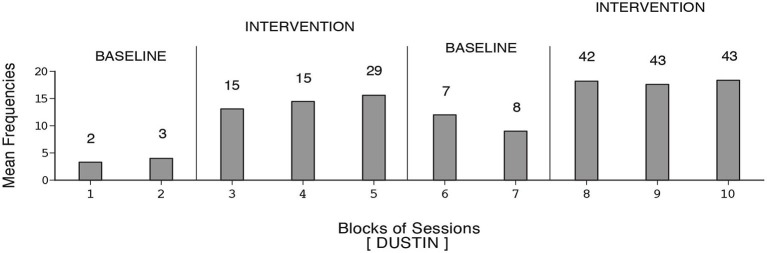
**Dustin’s data plotted as in Figure 1**.

**Figure 6:**
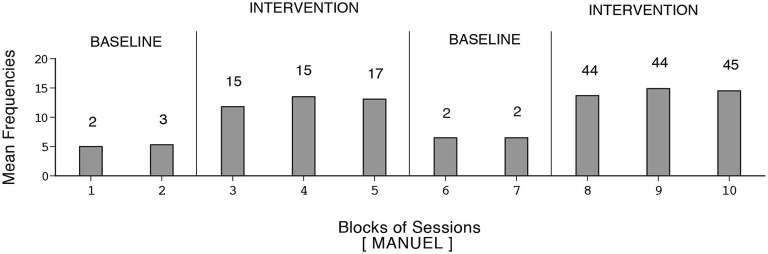
**Manuel’s data plotted as in Figure 1**.

The participants’ frequencies of independent responses (i.e., emitted without prompting from the research assistant) during the baseline and intervention phases are summarized in Figures 1–6. The bars indicate mean frequencies of responses per session over blocks of sessions. The number of sessions included in each block/bar is indicated by the numeral above it. To provide a clear picture of early response increases, the numbers of sessions included in the initial two bars of the first B phase are identical (i.e., 15) across participants, irrespective of their total numbers of sessions within that phase. Eunice’s mean frequency of responses was below six during the initial baseline, increased to about 13 during the first B phase, declined during the second baseline, and increased again to about 15 during the second B phase (i.e., 159 sessions). Gwen’s mean frequency was below five during the first baseline, exceeded 15 during the first B phase, declined during the second baseline, and exceeded 16 during the second B phase (i.e., 211 sessions). Coleen’s data resembled those of Eunice.

Harold’s mean frequency of responses was about four during the initial baseline, exceeded 12 during the first B phase, declined during the second baseline, and increased to nearly 14 during the second B phase (i.e., 109 sessions). Dustin’s response frequencies exceeded those of the other participants, reaching a mean of nearly 18 during the second B phase (i.e., 128 sessions). Also, his response decline during the second baseline was slow. Manuel’s mean frequency of responses was about five during the first baseline, increased to nearly 13 during the first B phase, declined during the second baseline, and increased to above 14 during the second B phase (i.e., 133 sessions). The differences between the first baseline and first B phase and between the second baseline and second B phase were fairly consistent and statistically significant (*p* < 0.01) on the Kolmogorov-Smirnov test (Siegel and Castellan, 1988) for all participants.

## Study II

### Method

#### Participants

The three participants (Adrian, Ben, and Vinnie) were in a rehabilitation center and had a diagnosis of E-MCS with pervasive motor impairment, lack of speech, and absence of any practical/functional skills, following brain injury and coma. Adrian was 51 years old, and had suffered rupture of aneurysm of the left middle cerebral artery with extended fronto-temporal hematoma and midline shift about 3 months prior to this study. His coma lasted about 3 weeks. Ben was 57 years old, and had suffered left pontine-mesencephalic hemorrhage of the brainstem due to cavernous malformation about 3 months prior to this study. His coma lasted about 3 weeks. Vinnie was 52 years old, and had been involved in a road accident, which resulted in diffuse axonal injury of the frontal and occipital bilateral regions and of the corpus callosum about 2 months prior to this study. Her coma lasted about 2 weeks.

At the start of the study, they were rated between the fifth and sixth level of the Rancho Levels of Cognitive Functioning (Hagen, 1998). All three were reported by staff and families to enjoy the presence of family members and friends talking to them. They were also observed to respond positively (by alerting, smiling, or vocalizing) to videos with music/songs, films, sport events, family members interacting with (talking to) them, and caregiver interventions such as refreshing/grooming or massaging (Lancioni et al., 2014b). Yet, they did not seem to have clear (reliable) understanding of verbal messages or simple pictorial images concerning (i.e., announcing or asking about) those particular stimulus events. They appeared quite interested in accessing (choosing among) those stimulus events. Their families had signed a consent form for the study, which had been approved by a scientific and ethics committee.

#### Position, stimulus events, and sessions

The participants sat in their wheelchair during all sessions of the study. Six or eight sets of 20 stimulus events were used, one per session in a rotation fashion. Each set involved (a) 11 videos with music and song scenes; (b) 3 videos with family members talking to them; (c) 3 videos illustrating caregiver interventions, such as refreshing/grooming; and (d) 3 videos with instruments producing distorted/blurred sounds. Music and songs, family members talking, and caregiver interventions were considered preferred events (see participants). Distorted/blurred sounds were considered non-preferred events and were interspersed with the others as a check on the participants’ choice purposefulness. Purposefulness was inferred if they showed general avoidance of the non-preferred events and high levels of responding to preferred events (Lancioni et al., 2011a, 2013e, 2014b).

#### Technology, response, and data recording

The technology involved a computer system with screen and sound amplifier that automatically presented the stimulus events available within the sessions and a microswitch that served to choose those events. The microswitch was (a) a mini shock-absorber device attached to the participant’s index finger (Adrian) that could be activated with a finger-tapping response; (b) an optic microswitch fixed on the participant’s right cheekbone, as described in Study I (Ben), that could be activated with prolonged eyelid closure; and (c) a flat box-like device fixed inside the participant’s hand (Vinnie) that could be activated with a hand-closure response (Lancioni et al., 2013b). The computer system presented a 5-s sample of each of the 20 stimulus events available in the session. During the baseline and the first 7–15 sessions of the intervention phase, the sample was accompanied by an attention-calling verbal expression such as “Want it?” or “Like it?” The expression was then made less audible and finally eliminated. Consequences for the participants’ responses (i.e., microswitch activations) were available only during the intervention sessions. If a response (microswitch activation) occurred within the 5 or 6 s that followed: (a) a music or song sample; (b) a family-member talking sample; and (c) a non-preferred stimulus sample, the system presented the matching stimulus for 20 s. If a response occurred within 5 or 6 s from a caregiver intervention sample (video illustration), the system called the caregiver who enacted that intervention for 20 s. If a response occurred within 5 or 6 s from the end of a 20-s stimulus presentation or caregiver intervention, the system provided another 20 s of the same stimulus or called the caregiver to reiterate the intervention. Absence of responding within 5 or 6 s from the end of (a) a sample; (b) a 20-s stimulus presentation; or (c) a caregiver intervention led the system to pause for about 10 s and then present the next sample of the sequence. One or two sessions per day took place. A session lasted until all stimulus samples had been presented. The samples (i.e., stimulus events) chosen and the 20-s stimulus presentations and caregiver interventions occurred per session were automatically recorded via the computer system.

#### Experimental conditions

The study was carried out according to a non-concurrent multiple baseline design across participants (Barlow et al., 2009). Adrian, Ben, and Vinnie had two, four, and five baseline sessions, respectively. The intervention phase included 48, 64, and 65 sessions for the three participants, respectively.

##### Baseline phase

The participants were provided with the microswitch for the finger, hand or eyelid response and the computer system. The system presented the samples of the stimulus events available and recorded the participants’ responses, as described above. However, those responses were never followed by 20-s stimulus presentations or caregiver interventions.

##### Intervention phase

During the intervention phase, the participants had the microswitch and the computer system, which worked as described in the *Technology, Response, and Data Recording* section. The 48–65 intervention sessions available for the participants were preceded by five practice sessions. During each of these sessions, the participants were led to respond to samples of positive stimulus events and after the end of 20-s stimulus presentations (i.e., to experience the consequences of their responding).

### Results

**Figure 7:**
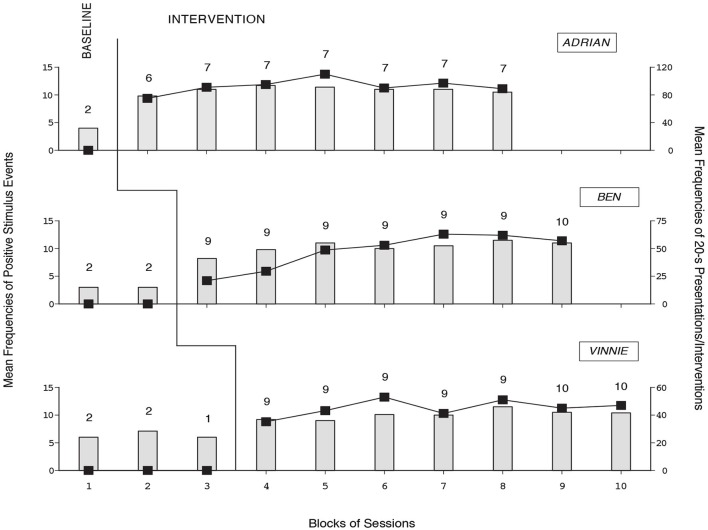
**The three panels summarize the data for Adrian, Ben, and Vinnie, respectively**. The bars and black squares indicate mean frequencies of positive stimulus events chosen per session and mean frequencies of 20-s stimulus presentations and caregiver interventions occurred per session, respectively, over blocks of sessions (with one exception; see Vinnie’s baseline). The number of sessions per bar-square combination is indicated by the numeral above it.

The three panels of Figure 7 summarize the data for Adrian, Ben, and Vinnie, respectively. The bars and black squares indicate mean frequencies of positive stimulus events chosen per session out of the 17 available (i.e., the music/song, family-member talking, and caregiver intervention events) and mean frequencies of 20-s stimulus presentations and caregiver interventions occurred per session, respectively, over blocks of sessions. The only exception is the last bar-square of Vinnie’ baseline that represents a single session. The number of sessions represented by each bar-square combination is indicated by the numeral above it.

During the baseline phase, the mean frequencies of positive stimulus events chosen were between three (Ben) and slightly above six (Vinnie). Choices did not have consequences, thus the mean frequencies of 20-s stimulus presentations and caregiver interventions were zero (see Figure 7). During the intervention phase, the participants’ mean frequencies of positive stimulus events chosen per session were about 10 or 11. The mean frequencies of 20-s stimulus presentations and caregiver interventions per session were 92, 48, and 45, respectively. Actually, the participants obtained several repetitions of various (highly preferred) stimuli/interventions by producing a response soon after they ended. The mean frequencies of non-preferred stimulus events chosen were close to zero during the baseline and intervention phases, suggesting purposeful choice behavior.

## Study III

### Method

#### Participants

The three participants (Harvey, Madelyn, and Lloyd) were in a rehabilitation center and had a diagnosis of E-MCS with pervasive motor impairment, lack of speech and absence of any practical/functional skills, following brain injury and coma. Harvey was 68 years old, and had suffered a left total circulation infarct stroke over 2 months prior to this study. His coma lasted about 1 week. Madelyn was 62 years old, and had been affected by viral meningo-encephalitis and Guillain-Barré syndrome about 6 years prior of this study. Her coma lasted about 2 weeks. Lloyd was 53 years old, and had suffered a fall causing multiple cranial fractures and diffuse axonal injury of the right temporal corpus callosum and mesencephalic regions about 10 months prior to this study. His coma lasted about 2 weeks.

At the start of the study, the participants’ conditions were rated between the sixth and seventh level of the Rancho Levels of Cognitive Functioning (Hagen, 1998). They understood verbal questions concerning their personal and family life and daily events, and could respond to them appropriately with head or eye movements. They showed interest in conversation and stimulation events as well as in work-related issues. They were observed to enjoy popular music/songs, film clips of sport and comedy, audio- and video-recordings of family members and friends talking to them, caregivers’ interventions (e.g., care and grooming procedures), and the reading of news or of history and religion passages (Lancioni et al., 2014b). They seemed eager to enter this study and access (choose among) the aforementioned options, and had agreed to it with affirmative head or eye responses. Their families had signed a consent form for the study, which had been approved by a scientific and ethics committee.

#### Position, sessions, and data recording

The participants sat in their wheelchair during all sessions. Sessions lasted 10 min or until the effects of any choice started before the 10-min limit had ended, and occurred 2–5 times a day. A choice involved two steps, that is, selecting (a) one of the general options available (e.g., songs, video-recordings of family members and friends, or caregiver interventions); and then (b) a specific variant of that option (see below). For each session, a research assistant recorded the frequencies of choices carried out, and specifically, of songs and film clips, audio- or video-recordings, caregiver interventions, and reading pieces activated/obtained. Interrater reliability was assessed in about 20% of the sessions, in which two research assistants recorded the choices simultaneously. Percentages of agreement (computed by dividing the number of sessions in which the two research assistants reported the same choices by the total number of sessions used for reliability and multiplying by 100) exceeded 90 for each participant.

#### Technology and response

The technology involved a computer system with screen and sound amplifier, and a microswitch. The computer system served to (a) show pictorial images of the choice options (i.e., songs, film clips, family and friends, caregiver interventions, and reading material) and their variants on its screen; (b) verbally identify and scan (illuminate) one of the images at a time; and (c) respond to microswitch activations. The microswitch technology used for the three participants involved (a) a small pressure device in front of the participant (Harvey) or inside the participant’s hand (Lloyd) that could be activated by hand-pressure or hand-closure responses, respectively; and (b) the Microsoft Kinect sensor with specific software (see Study I) that could be activated by prolonged eyelid closure (Madelyn).

#### Choice procedures

When the participants chose one of the options available (i.e., activated the microswitch while the image of such an option was being scanned), a new set of 5–8 pictorial images (i.e., option variants) appeared on the computer screen. Each of those variants (e.g., images of songs/singers or family and friends) was automatically verbalized and scanned. If the participants chose a song or film clip, the computer system played it for 1–2 min. If the participants chose a family member or friend, the computer system played the video- or audio-recording of that person talking to them for about 1 min. If the participants chose one of the reading items (e.g., a brief newspaper article or religious piece), the computer read it out in 1–2 min. If the participants chose one of the caregiver interventions (e.g., face washing), the computer system verbalized it so that the caregiver could carry it out. The participants could interrupt any stimulus event chosen at any time by activating the microswitch. After the choice of a stimulus event, the computer automatically reset the original screen with the original choice options (see above).

#### Experimental conditions

The study was carried out according to a non-concurrent multiple baseline design across participants (Barlow et al., 2009). Harvey had two baseline sessions while Madelyn and Lloyd had four baseline sessions. The intervention phase included 132, 125, and 110 sessions for the three participants, respectively.

##### Baseline phase

The participants were provided with a computer showing pictorial images of the choice options (i.e., songs, film clips, family and friends, caregiver interventions, and reading material) on its screen, and a mouse to activate those options. Lack of responding after 3–4 min led the research assistant to carry out a choice for them.

##### Intervention phase

During this phase, the full technology package (i.e., including the microswitch) was available and worked as described above. The intervention phase was preceded by six practice sessions in which the research assistant helped the participants carry out choices through the response sequences required (see *Choice Procedures*). Variation was ensured within the choice options (e.g., by using new songs or film clips).

### Results

**Figure 8:**
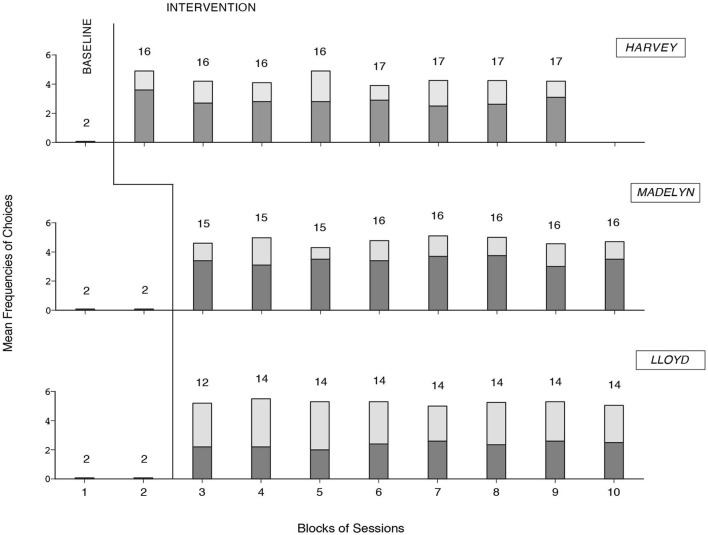
**The three panels summarize the data for Harvey, Madelyn, and Lloyd, respectively**. The bars in their entirety represent mean cumulative frequencies of choices made per session over blocks of sessions. The dark-gray sections of the bars represent mean frequencies for songs and videos together. The light-gray sections of the bars represent mean frequencies for audio- and video-recordings of family members, caregiver interventions, and reading items together. The number of sessions per block/bar is indicated by the numeral above it.

The three panels of Figure 8 summarize the data for Harvey, Madelyn, and Lloyd, respectively. The bars in their entirety represent mean cumulative frequencies of choices made per session over blocks of sessions. The dark-gray sections of the bars represent mean frequencies for songs and film clips together (i.e., leisure events). The light-gray sections of the bars represent mean frequencies for audio- and video-recordings of family members and friends, caregiver interventions, and reading items together (i.e., social and cultural events). The number of sessions included in each block/bar is indicated by the numeral above it.

During the baseline, the participants did not carry out any choice, as they could not use the mouse. During the intervention phase, all participants gained successful choice performance (i.e., activating the options available and specific variants of them, in line with their interests). The mean cumulative frequencies of choices made per session were above four for Harvey and Madelyn and above five for Lloyd. Choices concerning songs and film clips accounted for nearly two thirds of the choice totals for Harvey and Madelyn and less than half of the choice total for Lloyd.

## General discussion

The results of the three studies add new evidence in support of the beneficial impact of technology-aided intervention programs for post-coma persons with multiple disabilities (Lancioni et al., 2010, 2011a, 2012a,b, 2013a,d). Specifically, they show the relevance of those programs to allow (a) MCS participants to develop functional responses to access preferred environmental stimulation independently (Study I); and (b) E-MCS participants to choose among a variety of stimulus events automatically presented to them (Study II) or sought by them through specific response sequences (Study III). Based on previous literature in the area, one could argue that those programs constitute the best (perhaps the only) approach to date to help these participants achieve the aforementioned skills (Lombardi et al., 2002; Lancioni et al., 2010, 2014b,d; Conneeley, 2012; de Joode et al., 2012; Di Stefano et al., 2012; de Jong, 2013; Wall et al., 2013; Williamson et al., 2013). In light of the findings and of the technology used for the programs, a number of considerations may be forwarded.

First, the findings of Study I indicate that it is feasible to arrange an intervention strategy for MCS persons that does not simply enrich their environment and treat them as general stimulation recipients (Giacino, 1996; Vanier et al., 2002; Elliott and Walker, 2005; Georgiopoulos et al., 2010; Lotze et al., 2011). The data of Study I, in line with previous data in the area, indicate in fact that these persons can be helped to develop an active role with multiple responses, self-modulation of their stimulation input, and likely increases in alertness and attention (Taylor et al., 2007; Liberati and Birbaumer, 2012; Whyte and Nakase-Richarson, 2013; Lancioni et al., 2014b,c). The functional response and alertness increases observed during technology-aided sessions as opposed to stimulation sessions (see Lancioni et al., 2014c) stress the relevance of the former sessions/programs for promoting behavioral conditions essential for the rehabilitation/recovery process (Bagnato et al., 2013; de Jong, 2013; Eifert et al., 2013; Abbate et al., 2014; Pisa et al., 2014). Self-modulation (control) of the stimulation input may be instrumental to ensure a greater level of personal involvement and enjoyment compared to what might be expected from an externally regulated stimulation approach (Fischer et al., 2008; Munde et al., 2009; McDougall et al., 2010). With regard to this point, one could argue that the participants’ relatively rapid response increase was a sign of the highly motivating/reinforcing value of the stimulation they obtained and, indirectly, of their enjoyment of the sessions in general (Kazdin, 2001; Pierce and Cheney, 2008; Sunderland et al., 2009; Man et al., 2010; Catania, 2012).

Second, the responses selected for the participants of Study I were minimal movements of the eyelid, of the head or hand, which may be used for many persons with MCS and pervasive motor impairment. The microswitches employed for the responses included a simple optic sensor, a special touch pad, and the Kinect. The optic sensor is inexpensive and straightforward, but requires to be fixed in close connection with the area/response monitored. This may not always be desirable for participants who seem to dislike anything that touches them. For these participants, one may need microswitches that can monitor the responses from a distance rather than in contact with their body (Lancioni et al., 2013b). The Kinect is a tool that can be employed for this purpose (Galna et al., 2014; González-Ortega et al., 2014). Camera-based microswitches have also been used for the same purpose in the recent past (Lancioni et al., 2013b,c). The touch pad is a fairly sophisticated and highly functional device that can monitor minimal finger movements in a person who cannot move his or her hand (Lancioni et al., 2012a, 2013b). The cost of the last three types of microswitches is higher than that of typical microswitches and may range from around US $500 (the touch pad) to over US $1,000 (the Kinect). Obviously, the development of new microswitches and the control of their cost are critically important requirements for increasing the impact and acceptability of future intervention programs (Frankoff and Hatfield, 2011; Lindqvist and Borell, 2012; Posatskiy and Chau, 2012; Lancioni et al., 2013b, 2014b,d; Moghimi et al., 2013).

Third, Study II showed that E-MCS participants with pervasive motor disabilities, lack of speech, and uncertain receptive skills were empowered to choose among multiple leisure stimuli, as well as social and care stimuli, through a program presenting such stimuli automatically/directly. This program, which showed that the participants were purposeful in their choice behavior (i.e., they largely ignored negative stimuli), represents a clear step forward compared to the program used for MCS persons in Study I. Indeed, the participants of Study II managed to choose the stimuli or caregiver interventions that they wanted by responding to such stimuli/interventions (i.e., after a sample of them had been introduced or following the end of their previous presentation). Conversely, the same participants could bypass/reject the events that they did not want by simply abstaining from responding to those events. Their engagement time was not externally decided, but reflected their personal involvement and interest (Foley and Ferri, 2012). Higher levels of interest would lead them to produce higher levels of responses (choices) with consequent extension of the engagement time and vice versa (Pierce and Cheney, 2008). The technology for such a program involves a computer system with specific software and a microswitch. The use of such technology may be considered rather straightforward. Its cost of about US $2,000 may be affordable for many care and rehabilitation contexts (Hubbard Winkler et al., 2010; Dahlin and Rydén, 2011; Wallace, 2011).

Fourth, Study III showed the successful application of a choice program requiring higher initiative/communication skills and a wider range of interests on the side of the participants (i.e., as opposed to the program used in Study II). The participants were always to choose a specific stimulus or intervention option in order for the computer system to present the choice alternatives available within that option. Activation of one of the alternatives made it available for a relatively long time compared to the times used in Study II (i.e., except for caregiver interventions). The basic reason for this difference between studies can be summarized as follows. Study II was to help participants with limited initiative/communication to practice choice behavior and improve their level of activity and alertness. Study III was to help more advanced participants to experience a level of choice and choice consequences matching their condition and overall abilities (Lancioni et al., 2011b, 2013e). In line with their abilities, the program also allowed them to interrupt any stimulus event prematurely if they so desired. Aside from the differences, both studies/programs provided opportunities that the participants would not have had without the technology packages described. The technology used for Study III may be considered comparable to that used in Study II in terms of practicality and costs.

Fifth, the technology-based intervention programs reported in the three studies seem to represent plausible solutions for different groups of post-coma participants with multiple disabilities. Extending their use to additional individuals and upgrading (adapting) the technology components of the programs may be considered critical objectives of new research in this area. Positive replications of the beneficial impact of the programs are necessary to provide support for the generality of these results. Technology upgrades may start from the microswitch components of the programs (as mentioned above), with the aim of developing tools feasible for those individuals who find the present solutions less than satisfactory, due to a minimal behavioral repertoire or dislike for things touching them (Bauer and Elsaesser, 2012; Foley and Ferri, 2012; Gibson et al., 2012; Lancioni et al., 2012a, 2013b, 2014b; Posatskiy and Chau, 2012; Shih et al., 2012; Näslund and Gardelli, 2013; Seel et al., 2013). Improvements may also be considered within each of the computer systems. For example, the computer system used to regulate the delivery of stimulation contingent on the responses of MCS participants (i.e., Study I) could be set to determine the response frequencies under different types of stimuli and eventually change the stimuli used so as to maximize the participants’ response density and enjoyment (Fritz et al., 2013; Taheri et al., 2014).

In conclusion, the positive results obtained with the three studies provide additional evidence supporting the use of technology-aided programs to enhance the skills level (recovery process) of post-coma persons with multiple disabilities. Most immediate objectives of new research may be to confirm these results and improve the technology packages, as suggested above (Kennedy, 2005; Barlow et al., 2009; Goodwin, 2010). Other important goals may be to investigate (a) participants’ satisfaction with the programs (i.e., by including participants and programs like those of Studies II and III); and (b) families and staff’s opinions about the programs and suggestions for improving them (Lancioni et al., 2006; Callahan et al., 2008; Ripat and Woodgate, 2011; Lamontagne et al., 2013; Lenker et al., 2013; Lindstedt and Umb-Carlsson, 2013; Pouliquen et al., 2013).

## Conflict of interest statement

The authors declare that the research was conducted in the absence of any commercial or financial relationships that could be construed as a potential conflict of interest.
